# Rudolf Buchheim Award 2026: solanidine as a dietary derived CYP2D6 biomarker

**DOI:** 10.1007/s00210-026-05650-w

**Published:** 2026-07-03

**Authors:** Julian Peter Müller

**Affiliations:** Institute of Clinical Pharmacology, University Hospital RWTH Aachen, Wendlingweg 2, 52074 Aachen, Germany

**Keywords:** Solanidine, CYP2D6, Biomarker, Drug metabolism, Phenoconversion, Personalized medicine

## Abstract

The German Society for Experimental and Clinical Pharmacology and Toxicology (DGPT) is annually awarding the Rudolf Buchheim Award. This year the Rudolf Buchheim Award was awarded to Julian Peter Müller for outstanding work of a young researcher in the field of clinical pharmacology. He investigated the performance and applicability of the novel CYP2D6 biomarker solanidine. CYP2D6 is a genotypic and phenotypic highly variable drug-metabolizing enzyme involved in the metabolism of about 20% of commonly used drugs. Variability in CYP2D6 activity may lead to pronounced interindividual deviations in the pharmacokinetics of its drug substrates, potentially leading to adverse events or therapy failure. CYP2D6 genotyping allows to predict metabolizer phenotypes, e.g., poor, intermediate, normal or ultrarapid, but cannot take into account additional variation due to co-medication or other factors. In the awarded work he could demonstrate that a single plasma sample is sufficient for accurate CYP2D6 phenotyping, and that by using solanidine, CYP2D6 phenoconversion, a genotype–phenotype mismatch, due to polypharmacy can be elucidated. The use of the CYP2D6 biomarker solanidine allows to study non-genetic variation of CYP2D6 activity and opens up new possibilities in personalized medicine. Application of solanidine phenotyping may help to understand factors of CYP2D6 phenoconversion and may be applied with patients to optimize therapy decisions and to improve outcomes and safety.

## Short communication

Cytochrome P450 2D6 (CYP2D6) is one of the most relevant drug-metabolizing enzymes demonstrating pronounced genotypic and phenotypic variability. Around 20% of commonly used drugs are metabolized by CYP2D6 (Taylor et al. 2020). CYP2D6 variability is crucial for therapy efficacy and adverse drug reactions. Due to additional non-genetic factors on CYP2D6 activity, such as drug-drug interactions, genotyping may not be sufficient to accurately predict CYP2D6 activity in patients. The preemptive phenotyping of CYP2D6 activity may improve therapy outcomes and safety beyond CYP2D6 genotyping.

**Fig. 1:**
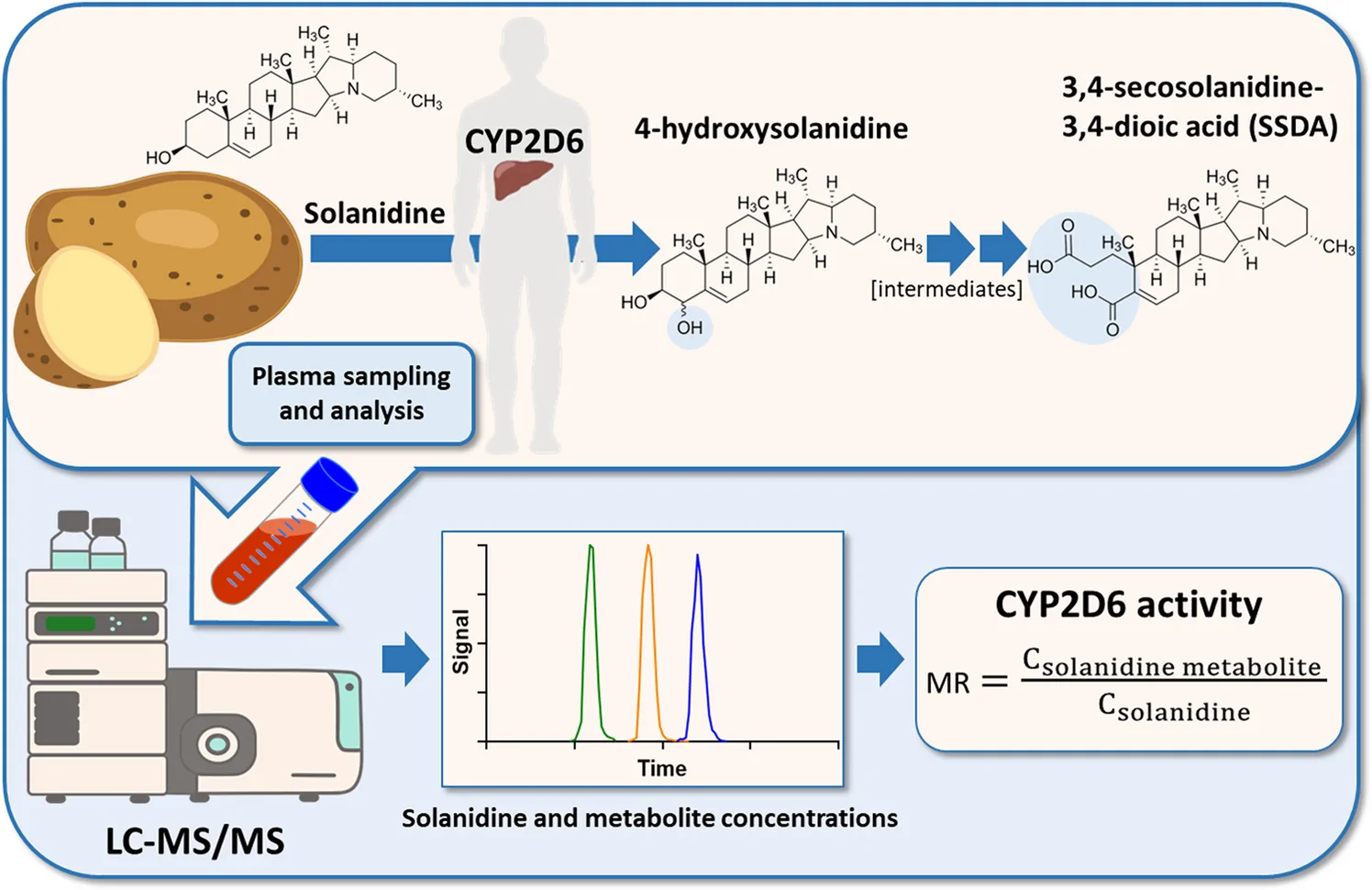
Solanidine is taken up by dietary consumption of potatoes and potato-derived products. In the liver, solanidine is metabolized by CYP2D6 to 4-hydroxysolanidine and through intermediate steps to 3,4-secosolanidine-3,4-dioic acid (SSDA). After potato consumption, solanidine and its metabolites can be measured by liquid chromatography coupled to tandem mass spectrometry (LC–MS/MS) in plasma samples. Dividing the solanidine metabolite plasma concentrations by solanidine plasma concentrations results in metabolic ratios (MR) that were able to accurately depict the CYP2D6 phenotype and to identify genotype-predicted CYP2D6 poor metabolizers

The recent identification of solanidine and its metabolites as CYP2D6 biomarkers opens new possibilities in personalized medicine and the study of variability in drug metabolism (Tay-Sontheimer et al. 2014; Magliocco et al. 2021). Solanidine is a steroidal alkaloid derived from potatoes and is taken up from the diet; it is specifically metabolized by CYP2D6 to 4-hydroxysolanidine and via intermediates to 3,4-secosolanidine-3,4-dioic acid (SSDA) (Magliocco et al. 2021; Behrle et al. 2022) (Fig. 1). Metabolic ratios of 4-hydroxysolanidine/solanidine were able to identify CYP2D6 poor metabolizers with high precision and the metabolic ratio of SSDA/solanidine showed strong correlations to the metabolism of CYP2D6 substrates risperidone, dextromethorphan, and tamoxifen and was sensitive to CYP2D6 inhibition (Magliocco et al. 2021; Medwid et al. 2024; Wollmann et al. 2023; Wollmann et al. 2023).

The 2026 Rudolf Buchheim Award was awarded to Julian Peter Müller working on solanidine phenotyping together with the team of the Institute of Clinical Pharmacology at the University Hospital RWTH Aachen under the supervision of Professor Julia C. Stingl and Professor Katja S. Just.

In the first study, we addressed the correlation of solanidine metabolic ratios with the metabolic ratio of the commonly used CYP2D6 probe drug metoprolol. In a CYP phenotyping cocktail study with 20 participants, which was performed in close cooperation with the Institute for Occupational, Social and Environmental Medicine at the University Hospital RWTH Aachen, 12.5 mg metoprolol among other probe drugs was orally administered to the participants and blood plasma sampling was performed over 24 h. Solanidine and its metabolites were measured by liquid chromatography tandem mass spectrometry (LC–MS/MS) in 5 plasma samples of each participant over the study day (0, 2.5, 5, 8, and 24 h) and the resulting metabolic ratios were correlated to the CYP2D6 phenotype given as the metabolic ratio of the area under the curve (AUC) of α-hydroxymetoprolol and metoprolol. We could show that SSDA/solanidine metabolic ratios at each of the single plasma sample measurements over the study day correlated significantly with the metabolic ratio of the AUC of α-hydroxymetoprolol/metoprolol, which demonstrated high stability of the solanidine metabolic ratios and that single plasma samples are sufficient for CYP2D6 phenotyping (Müller et al. 2024).

In the second study, we applied CYP2D6 phenotyping by solanidine metabolic ratios to a cohort of geriatric polypharmacy patients with a median age of 83 and a median intake of 15 drugs (Sarömba et al. 2025). The full medication intake and the CYP2D6 genotype of these patients were assessed, and the genotype-derived CYP2D6 activity score was assigned. We measured solanidine and its metabolites in a single plasma sample of each of the 88 patients by LC–MS/MS and formed the metabolic ratios. By calculating a shift in CYP2D6 activity score, we were able to include the CYP2D6 genotype information and assessed the deviation of the individual patients solanidine metabolic ratios from the linear regression equation of solanidine metabolic ratios against the genotype-predicted CYP2D6 activity score. With this approach, we were able to demonstrate a stronger downward shift in activity score with an increasing intake of CYP2D6 interacting medications (substrates and inhibitors), suggesting a phenoconversion of CYP2D6 (Sarömba et al. 2025).

Previous studies and our work demonstrate that CYP2D6 phenotyping by solanidine metabolic ratios shows a huge potential in identifying and studying non-genetic factors of CYP2D6 variability. Solanidine phenotyping may be applied to larger well-described cohorts to address yet unknown factors of CYP2D6 variability. In addition, solanidine phenotyping has the potential to improve our ability to predict CYP2D6 activity in the clinical setting and thereby to facilitate tailored drug therapies.

## Data Availability

No data was used for this article.
